# Aprocitentan: New insights

**DOI:** 10.3389/fcvm.2022.1093406

**Published:** 2022-12-22

**Authors:** Fahimeh Varzideh, Urna Kansakar, Stanislovas S. Jankauskas, Gaetano Santulli

**Affiliations:** ^1^Department of Medicine, Division of Cardiology, Einstein-Mount Sinai Diabetes Research Center (ES-DRC), Wilf Family Cardiovascular Research Institute, Albert Einstein College of Medicine, New York, NY, United States; ^2^Department of Molecular Pharmacology, Einstein Institute for Aging Research, Einstein Institute for Neuroimmunology and Inflammation, Fleischer Institute for Diabetes and Metabolism (FIDAM), Albert Einstein College of Medicine, New York, NY, United States

**Keywords:** ACT-132577, aprocitentan, blood pressure, clinical trial, endothelin, hypertension, PRECISION, resistant hypertension

Resistant hypertension is a condition observed in >10% of hypertensive patients, defined by blood pressure (BP) targets not achieved despite treatment with at least three anti-hypertensive medications of different classes, including a diuretic, a blocker of the renin-angiotensin system, and a long-acting calcium channel blocker (1). Current guidelines recommend the use of spironolactone as the preferred 4th line drug, with a better BP lowering efficacy compared to adrenergic α-blockers or β blockers. Notably, a major component in the pathophysiology of hypertension, i.e., the endothelin (ET) system, has been somehow overlooked in the management of resistant hypertension. Indeed, while initial studies with the ET receptor antagonists bosentan and darusentan had shown a BP lowering effect in hypertensive patients (2–4), a subsequent trial testing darusentan as an add-on therapy in resistant hypertension did not confirm these findings (5).

ETs are 21-amino-acid vasoconstricting peptides mainly produced by endothelial cells; three endogenous isoforms are known (ET-1, ET-2, and ET-3), which exert their actions by binding two main types of receptors (A and B) (6, 7).

Aprocitentan (ACT-132577) is an antagonist that prevents the binding of ET to both types of receptors (8). Aprocitentan is a member of the class of sulfamides in which one of the amino groups of sulfonamide has been substituted by a 5-(4-bromophenyl)-6-{2-[(5-bromopyrimidin-2-yl)oxy]ethoxy}pyrimidin-4-yl group (Figure 1). Studies assessing its pharmacokinetics have determined that aprocitentan has a half-life of ~44 h and steady-state conditions are reached after 8 days (9–11).

**Figure 1:**
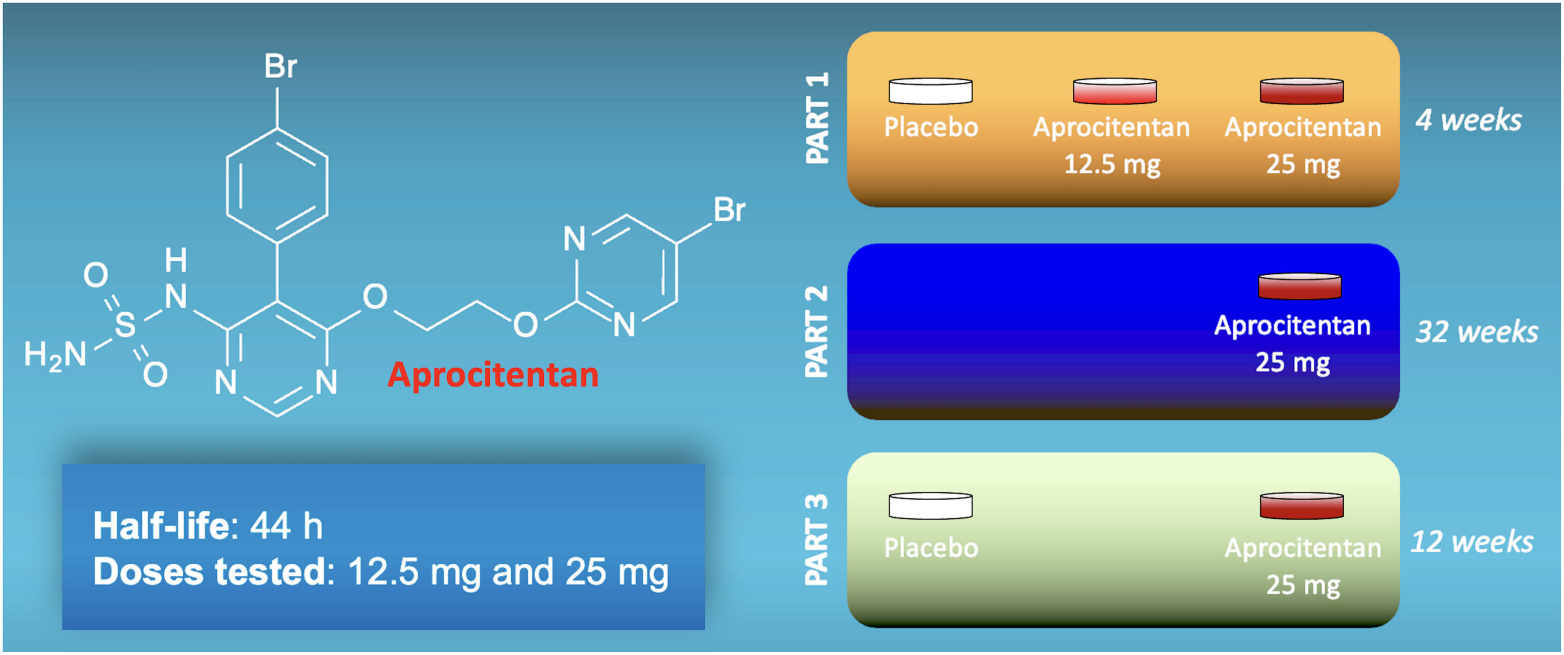
Structure of aprocitentan and schematic representation of the three parts of the PRECISION trial.

Following promising results in preclinical settings (8), aprocitentan has been tested in a multicenter, blinded, randomized, PaRallEl-group, Phase 3 study with aproCItentan in Subjects with ResIstant HypertensiON (PRECISION, Clinicaltrials.gov registration ID: NCT03541174). The results of the trial have been presented by Dr. Marcus P. Schlaich at the latest Scientific Sessions of the American Heart Association in Chicago (Session “Late-Breaking Science: Resistant Hypertension: A Pressure Cooker”) and simultaneously published in *The Lancet* (12).

The PRECISION trial was conducted from 18 June 2018 to 25 April 2022, screening 1965 individuals at 193 sites in 22 countries, and 730 were randomly assigned. The design of the study included a 12-week screening period to exclude pseudo-resistant hypertension by changing all patients to a standardized single-pill triple-therapy (amlodipine, valsartan, hydrochlorothiazide) at fixed doses, a 4-week double-blind, placebo-controlled treatment phase (part 1, double-blind, completed by 704 patients); a 32-week single-blind, active-treatment phase (part 2, single-blind, completed by 613 patients); and a 12-week double-blind, placebo-controlled withdrawal phase (part 3, double-blind re-randomization, completed by 577 patients). The main characteristics were similar across all treatment groups at baseline. Black patients represented 11% of all participants (37% of the participants from the United States), more than 60% of enrolled patients were taking more than four anti-hypertensive drugs at enrollment, ~20% had history of heart failure, and 52–56% had history of diabetes.

The results of the PRECISION trial are straightforward: 4 weeks after treatment initiation (primary outcome), the least square mean difference changes vs. placebo of unattended automated office systolic BP were −3.8 ± 1.3 mm Hg (97.5% CI: −6.8 to −0.8, = 0.0042) for aprocitentan 12.5 mg, and −3.7 ± 1.3 mm Hg (−6.7 to −0.8, *p* = 0.0046) for aprocitentan 25 mg. Office diastolic BP decreased as well-compared to placebo (−3.9 mm Hg, 95% CI: −5.6 to −2.3 for aprocitentan 12.5 mg; −4.5 mm Hg, 95% CI: −6.1 to −2.9 for aprocitentan 25 mg). BP was maintained during part 2, while in part 3, after 4 weeks of withdrawal, a significant increase was detected in the placebo arm compared to aprocitentan in terms of both systolic (+5.8 mm Hg, 95% CI: 3.7–7.9, *p* < 0.0001) and diastolic BP (+5.2 mm Hg, 95% CI: 3.8–6.6, *p* < 0.0001). The results from office measurements were confirmed by ambulatory BP monitoring across the 24-h period, thereby providing robust data on short- and long-term safety and efficacy of the dual ET receptor antagonist aprocitentan.

A significant antiproteinuric effect was also observed at both doses of aprocitentan, which is relevant and truly promising in terms of reduction of target organ damage; further studies examining in detail cardiovascular parameters are warranted to corroborate the role of aprocitentan in this sense. Interestingly, subgroup analyses revealed statistically significant beneficial effects on BP of 25 mg aprocitentan in elderly subjects (≥75-year-old), in patients with estimated glomerular filtration rate (eGFR) between 15 and 60 ml/min/1.73 m^2^, and individuals with urine albumin-creatinine ratio (UACR) > 300 mg/g, all characteristics that tend to be associated with difficult-to-control hypertension.

One of the most common side effects of ET antagonists is represented by fluid retention, which was observed also in this trial: 9.1% of patients in the low dose aprocitentan and 18.4% in the high dose; these events were clinically manageable with the addition or up-titration of diuretic therapy, causing discontinuation of treatment only in one case. This adverse event, although being relatively controllable with diuretics, may pose potentially serious risks in patients with heart failure and/or chronic kidney disease. In this sense, it is important to note that patients with severe hypertension (grade 3) were excluded from the PRECISION trial, as well as subjects with NYHA III–IV heart failure, and patients with major cardiovascular, renal, and cerebrovascular complications in the 6 months preceding the enrollment.

The PRECISION trial is not exempt from limitations, including the lack of a placebo control in the single-blind part 2 of the study; in this sense, we would also like to emphasize the substantial BP lowering effect of placebo in the double-blind part 1, which might be attributable to the attentive management of patients with resistant hypertension, as seen with the so-called therapeutic concordance (1), in the setting of a clinical trial. Moreover, a direct comparison with spironolactone is not provided, and the actual clinical relevance of the results of the PRECISION trial remains to be determined. For instance, future studies with a long-term follow-up evaluating the effects of aprocitentan on cardiovascular outcomes and target-organ damage are warranted.

In conclusion, the dual ET antagonism obtained with aprocitentan, based on the solid rationale of targeting a currently unopposed pathobiological pathway of hypertension, represents a new alternative approach to treat resistant hypertension.

## Author contributions

All authors listed have made a substantial, direct, and intellectual contribution to the work and approved it for publication.

## References

[1] Trimarco V Izzo R Mone P Lembo M Manzi MV Pacella D. Therapeutic concordance improves blood pressure control in patients with resistant hypertension. Pharmacol Res. (2023) 187:106557. 10.1016/j.phrs.2022.106557 36402254 PMC9943685

[2] Weber MA Black H Bakris G Krum H Linas S Weiss R. A selective endothelin-receptor antagonist to reduce blood pressure in patients with treatment-resistant hypertension: a randomised, double-blind, placebo-controlled trial. Lancet. (2009) 374:1423–31. 10.1016/S0140-6736(09)61500-2 19748665

[3] Krum H Viskoper RJ Lacourciere Y Budde M Charlon V. The effect of an endothelin-receptor antagonist, bosentan, on blood pressure in patients with essential hypertension. Bosentan Hypertension Investigators. N Engl J Med. (1998) 338:784–90. 10.1056/NEJM199803193381202 9504938

[4] Black HR Bakris GL Weber MA Weiss R Shahawy ME Marple R. Efficacy and safety of darusentan in patients with resistant hypertension: results from a randomized, double-blind, placebo-controlled dose-ranging study. J Clin Hypertens. (2007) 9:760–9. 10.1111/j.1524-6175.2007.07244.x 17917503 PMC8110158

[5] Bakris GL Lindholm LH Black HR Krum H Linas S Linseman JV. Divergent results using clinic and ambulatory blood pressures: report of a darusentan-resistant hypertension trial. Hypertension. (2010) 56:824–30. 10.1161/HYPERTENSIONAHA.110.156976 20921430

[6] Speck D Kleinau G Szczepek M Kwiatkowski D Catar R Philippe A. Angiotensin and endothelin receptor structures with implications for signaling regulation and pharmacological targeting. Front Endocrinol. (2022) 13:880002. 10.3389/fendo.2022.880002 35518926 PMC9063481

[7] Dhaun N Webb DJ. Endothelins in cardiovascular biology and therapeutics. Nat Rev Cardiol. (2019) 16:491–502. 10.1038/s41569-019-0176-3 30867577

[8] Trensz F Bortolamiol C Kramberg M Wanner D Hadana H Rey M. Pharmacological characterization of aprocitentan, a dual endothelin receptor antagonist, alone and in combination with blockers of the renin angiotensin system, in two models of experimental hypertension. J Pharmacol Exp Ther. (2019) 368:462–73. 10.1124/jpet.118.253864 30622171

[9] Sidharta PN Melchior M Kankam MK Dingemanse J. Single- and multiple-dose tolerability, safety, pharmacokinetics, and pharmacodynamics of the dual endothelin receptor antagonist aprocitentan in healthy adult and elderly subjects. Drug Des Devel Ther. (2019) 13:949–64. 10.2147/DDDT.S199051 30962677 PMC6435120

[10] Verweij P Danaietash P Flamion B Menard J Bellet M. Randomized dose-response study of the new dual endothelin receptor antagonist aprocitentan in hypertension. Hypertension. (2020) 75:956–65. 10.1161/HYPERTENSIONAHA.119.14504 32063059 PMC7098434

[11] Fontes MSC Dingemanse J Halabi A Tomaszewska-Kiecana M Sidharta PN. Single-dose pharmacokinetics, safety, and tolerability of the dual endothelin receptor antagonist aprocitentan in subjects with moderate hepatic impairment. Sci Rep. (2022) 12:19067. 10.1038/s41598-022-22470-z 36352054 PMC9645340

[12] Freeman MW Halvorsen YD Marshall W Pater M Isaacsohn J Pearce C. Phase 2 trial of baxdrostat for treatment-resistant hypertension. N Engl J Med. (in press). 10.1056/NEJMoa2213169 36342143

